# Supplementary material for: In Silico Analysis of a Drosophila Parasitoid Venom Peptide Reveals Prevalence of the Cation–Polar–Cation Clip Motif in Knottin Proteins
Source: Pathogens. 2023 Jan 14;12(1):143. doi: 10.3390/pathogens12010143 (PMC9865768; doi:10.3390/pathogens12010143)
Supplement: Supplementary file 1 [file pathogens-12-00143-s001.zip › pathogens-2132839-SM.pdf]

**Table S1.** Identified homologs of LhKNOT using different sequence-based approaches.

| **Name of Organism (Genus & Species)** | **Accession # (Database)** | **% Identity** | **% Similarity** | **E-Value** | **Query Coverage** | **Method of Identification** |
|---|---|---|---|---|---|---|
| Common Ice Plant (*Mesembryanthemum crystallinum)* | AAC19399 (NCBI nr) | 42.59% | 64% | 2e-07 | 88% | BLASTp (nr) PSI-BLAST |
| Wheat Curl Mite (*Aceria tosichella)* | MDE48292.1 (NCBI nr) | 33.90% | 54% | 3e-04 | 93% | BLASTp (nr) BLASTp (TSA) |
| Cowpea Seed Beetle (*Callosobruchus maculatus)* | VEN35376.1 (NCBI nr) | 36.84% | 61% | 9e-09 | 93% | PSI-BLAST BLASTp (nr) HMMER |
| Red Mites (*Dinothrombium tinctorium)* | RWS16713.1 (NCBI nr) | 40.98% | 57% | 6e-08 | 100% | PSI-BLAST BLASTp (nr) HMMER |
| Parasitoid Wasp (*Trichogramma pretiosum)* | XP_014233229.1 (NCBI nr) | 54.72% | 64% | 7e-08 | 86% | PSI-BLAST |
| Click Beetle (*Ignelater luminosus)* | KAF2884324.1 (NCBI nr) | 41.38% | 58% | 6e-07 | 95% | PSI-BLAST |
| Parasitoid Wasp (*Trichogramma brassicae)* | CAB0031014.1 (NCBI nr) | 56.25% | 64% | 1e-06 | 78% | PSI-BLAST BLASTp (nr) HMMER |
| Common Pollen Beetle (*Brassicogethes aeneus)* | CAH0563829.1 (NCBI nr) | 43.10% | 55% | 9e-06 | 95% | PSI-BLAST |

| | | | | | | |
|---|---|---|---|---|---|---|
| Ash Borer (*Agrilus planipennis)* | XP_018321119.1 (NCBI nr) | 40.98% | 59% | 8e-05 | 100% | PSI-BLAST HMMER |
| Sawfly (*Neodiprion lecontei)* | XP_015517007.2 (Uniprot) | 37.70% | 57% | 6e-04 | 100% | PSI-BLAST HMMER |
| Fungus (*Akanthomyces lecanii)* | A0A168C5L6 (Uniprot) | 41.00% | 56.40% | 7.6e-11 | 63% | HMMER |
| Fungus (*Cordyceps Javanica)* | A0A545VVU1 (Uniprot) | 43.60% | 56.40% | 1.1e-07 | 63% | HMMER |
| Fungus (*Cordyceps confragosa)* | A0A179IJT7 (Uniprot) | 41.00% | 56.40% | 3.7e-07 | 63% | HMMER |
| Fungus (*Rosellinia necatrix)* | A0A1S8A723 (Uniprot) | 44.00% | 50.00% | 1.1e-06 | 81.6% | HMMER |
| Fungus (*Beauveria bassiana)* | A0A0A2VUF6 (Uniprot) | 43.20% | 56.80% | 4.8e-06 | 60% | HMMER |
| Harlequin Beetle (*Acrocinus longimanus)* | 1Q3J (PDB) | 51.52% | 66.00% | 6e-05 | 53% | BLASTp (PDB) |
| Leafy Cactus (*Pereskia bleo)* | 5XBD (PDB) | 40.00% | 51.00% | 8e-05 | 58% | BLASTp (PDB) |

**Table S2.** Putative *L. heterotoma* homologs of LhKNOT. Sequences were identified via tblastn from three Lh14 transcriptome datasets (see Methods for accession numbers). Transcripts grouped by shaded cells encode identical knottin peptides. Transcript accession numbers shown in bold encode two distinct peptides. Results were retrieved from the Description Table available from NCBI tblastn results.

| NCBI accession number | % Identity | E-Value | % Query Coverage |
|---|---|---|---|
| GHUQ01074597.1 | 47.06 | 0.8 | 53.00 |
| GHUQ01047008.1 | 37.50 | 0.58 | 53.00 |
| GHUP01112279.1 | 48.33 | 3.00E-15 | 100.00 |
| **GHUP01112276.1** | 49.15 | 8E-15 | 100.00 |
| GHUQ01062822.1 | 48.33 | 2E-14 | 100.00 |
| GHUQ01062810.1 | 49.15 | 6E-15 | 98.00 |
| GHUP01112278.1 | 48.33 | 1E-14 | 100.00 |
| **GHUP01112277**.1 | 49.15 | 2.00E-14 | 100.00 |
| **GHUP01112276.1** | 49.15 | 8E-15 | 100.00 |
| **GHUP01112277.1** | 49.15 | 2.00E-14 | 100.00 |
| GAJC01013378.1 | 76.32 | 1E-13 | 63.00 |
| GHUQ01006582.1 | 76.32 | 2E-13 | 63.00 |
| GHUQ01006580.1 | 78.95 | 1E-14 | 63.00 |
| GHUQ01006581.1 | 78.95 | 2E-14 | 63.00 |
| GHUP01140860.1 | 78.95 | 4E-14 | 63.00 |
| GHUP01140857.1 | 78.95 | 5E-14 | 63.00 |
| GAJC01011443.1 | 65.85 | 1E-11 | 68.00 |
| GHUQ01051051.1 | 65.85 | 5E-11 | 68.00 |
| GHUP01089975.1 | 51.67 | 7E-17 | 100.00 |
| GHUQ01026197.1 | 56.14 | 2E-16 | 95.00 |
| GHUP01112307.1 | 55.00 | 1E-14 | 100.00 |
| GHUQ01062808.1 | 75.00 | 7E-23 | 100.00 |
| GHUP01093382.1 | 66.67 | 1E-22 | 100.00 |

| | | | |
|---|---|---|---|
| GHUP01093384.1 | 66.67 | 3E-22 | 100.00 |
| GHUP01011997.1 | 35.94 | 0.006 | 100.00 |
| GAJC01018499.1 | 48.72 | 0.000009 | 63.00 |
| GHUQ01075921.1 | 48.72 | 0.00002 | 63.00 |
| GHUP01000498.1 | 48.72 | 0.00004 | 63.00 |
| GHUP01000497.1 | 48.72 | 0.00004 | 63.00 |
| GAJC01009090.1 | 44.44 | 0.004 | 58.00 |
| GHUQ01090262.1 | 44.44 | 0.013 | 58.00 |
| GHUP01053005.1 | 44.44 | 0.025 | 58.00 |

**Table S3.** Putative *L. boulardi* knottin homologs of LhKNOT. Sequences were identified via tblastn from four *L. boulardi* transcriptome datasets (see Methods for accession numbers). Identical knottin peptides derived from different transcripts are grouped by color. Results were retrieved from the Description Table from NCBI tblastn results.

| NCBI accession # | % Identity | E-value | % Query Coverage |
|---|---|---|---|
| GAJA01018727.1 | 53.33 | 5.00E-16 | 100 |
| GISX01146955.1 | 53.33 | 3.00E-15 | 100 |
| GITC01024163.1 | 53.33 | 6.00E-15 | 100 |
| GAJA01018424.1 | 38.33 | 1.00E-07 | 100 |
| GISX01025604.1 | 38.33 | 4.00E-07 | 100 |
| GISX01025592.1 | 38.33 | 5.00E-07 | 100 |
| GITC01178462.1 | 38.33 | 0.000002 | 100 |
| GITC01178474.1 | 38.33 | 0.000002 | 100 |
| GITC01178472.1 | 38.33 | 0.000002 | 100 |
| GITC01178467.1 | 38.33 | 0.000002 | 100 |
| GITC01178471.1 | 38.33 | 0.000002 | 100 |
| GITC01178466.1 | 38.33 | 0.000002 | 100 |
| GITC01178468.1 | 38.33 | 0.000002 | 100 |
| GAJA01013374.1 | 43.86 | 1.00E-11 | 95 |
| GAJA01001863.1 | 63.64 | 2.00E-07 | 53 |
| GGGI01004962.1 | 63.64 | 5.00E-07 | 53 |
| GISX01146961.1 | 56.67 | 2.00E-16 | 100 |
| GISX01025601.1 | 56.1 | 2.00E-10 | 68 |
| GISX01146968.1 | 64.1 | 6.00E-12 | 65 |
| GISX01046436.1 | 44.74 | 0.012 | 60 |
| GISX01016500.1 | 33.33 | 0.16 | 60 |
| GITC01073258.1 | 33.33 | 0.28 | 60 |
| GISX01112026.1 | 48.65 | 0.027 | 58 |

| | | | |
|---|---|---|---|
| GITC01104371.1 | 48.65 | 0.049 | 58 |
| GGGI01012121.1 | 48.65 | 0.007 | 58 |
| GISX01151661.1 | 41.67 | 0.065 | 58 |
| GISX01151658.1 | 42.42 | 0.038 | 53 |
| GITC01143021.1 | 42.42 | 0.089 | 53 |
| GISX01151653.1 | 47.22 | 0.086 | 58 |
| GITC01143015.1 | 48.48 | 0.19 | 53 |
| GISX01112051.1 | 43.24 | 0.45 | 58 |
| GITC01104365.1 | 43.24 | 0.82 | 58 |
| GGGI01006767.1 | 50 | 0.19 | 41 |
| GISX01121040.1 | 52.78 | 2.00E-07 | 56 |
| GITC01206261.1 | 52.78 | 4.00E-07 | 56 |
| GISX01121036.1 | 52.78 | 5.00E-07 | 56 |
| GISX01121038.1 | 52.78 | 0.00002 | 56 |
| GITC01206267.1 | 52.78 | 0.00003 | 56 |
| GISX01056433.1 | 63.64 | 0.000001 | 53 |
| GITC01031425.1 | 63.64 | 0.000002 | 53 |
| GISX01112052.1 | 50 | 0.7 | 41 |
| GITC01044290.1 | 43.1 | 5.00E-11 | 96 |
| GITC01089342.1 | 68.42 | 5.00E-12 | 63 |
| GITC01142054.1 | 45.95 | 0.061 | 58 |
| GITC01158992.1 | 44.12 | 0.012 | 55 |
| GGGI01003630.1 | 71.05 | 9.00E-13 | 63 |

**Table S4.** Putative knottin sequences from *Ganaspis* spp., homologous to LhKNOT. *G. hookeri* sequences were identified with tblastn, while *G. brasiliensis* sequences were identified with blastp. Results for *G. hookeri* and *G. brasiliensis* were obtained from the Description Table available from NCBI search results. Shaded sequences encode identical peptides.

| **Organism, NCBI accession number** | **% Identity** | **E-value** | **% Query Coverage** |
|---|---|---|---|
| *G. hookeri* GAIW01014505.1 | 65.85 | 7E-13 | 68 |
| *G. hookeri* GAIW01010507.1 | 42.5 | 0.002 | 65 |
| *G. hookeri* GAIW01021714.1 | 35.71 | 0.049 | 68 |
| *G. hookeri* GAIW01004791.1 | 39.02 | 0.084 | 65 |
| *G. hookeri* GAIW01013268.1 | 36.59 | 0.087 | 66 |
| *G. hookeri* GAIW01013267.1 | 36.59 | 0.088 | 66 |
| *G. hookeri* GAIW01013559.1 | 35.9 | 0.29 | 63 |
| *G. brasiliensis* g19460.t1 | 41.27 | 1E-06 | 93 |
| *G. brasiliensis* g14381.t1 | 35.71 | 0.007 | 65 |

**Table S5**. A list of amino acid residues in the CPC clips of examined known knottin, modeled knottin peptides, and Drosomycin. Distance measurements (Å) of alpha-carbon and sidechain center of mass (via pseudo-atom) of participating residues of identified CPC Clip motifs are shown. Key: + Known offensive knottins (toxins), Φ *in silico* models, * non-knottin fold, ± alternate putative peptide, highlighted in green and yellow to demarcate the peptides arising from the same transcript. The CPC clip residues and measurements are based on top docking scenario of interaction with heparin.

| Species Name | Protein (PDB ID or Accession #) | Function | Participating Residues (C-P-C′) | Alpha Carbons [R-Group Center of Mass] (CC′-CP-C′P) | Reference |
|---|---|---|---|---|---|
| *Leptopilinia heterotoma* | LhKNOT [*L. heterotoma* knottin 1] (GAJC01011813.1; GHUP01093383.1; GHUQ01062826.1; GHUQ01062814.1; GHUQ01062818.1) | Venom Peptide | R1-S3-R14 | 14.3Å - 6.2Å - 13.7Å [12.9Å - 7.8Å - 13.2Å] | [1] |
| *Acrocinus longimanus* | Alo-3 (PDB ID:1Q3J) | Antimicrobial Peptide | K3-Y32-R35 | 7.7Å - 3.8Å - 10.3Å [9.4Å - 5.5Å - 10.6Å] | [2] |
| *Phytolacca americana* | PAFP-S (PDB ID:1DKC) | Antifungal Peptide | K5-N6-R38 | 9.8Å – 6.9Å – 8.9Å [10.6Å - 6.8Å - 14.0Å] | [3] |
| *Pereskia bleo* | pB1 (PDB ID:5XBD) | Wound-Healing, Anti-*Candida* | R24-Y25-R35 | 14.8Å - 3.8Å - 15.5Å [16.3Å - 6.2Å - 13.8Å] | [4] |
| *Agelenopsis aperta* | Omega-agatoxin-IVA (PDB ID:1IVA) | Neurotoxin ($Ca^{2+}$ channel)+ | K13-T17-R23 | 11.5Å - 7.0Å - 11.3Å [13.4Å - 5.8Å - 11.1Å] | [5] |

| | | | | | |
|---|---|---|---|---|---|
| *Agelenopsis aperta* | Omega-Agatoxin-IVB (PDB ID:1AGG) | Neurotoxin ($Ca^{2+}$ channel) + | R39-S46-R21 | 11.2Å – 4.8Å – 7.7Å [11.5Å – 4.4Å – 8.3Å] | [5] |
| *Hadronyche verusta* | Atracotoxin-HV1 (PDB ID:1AXH) | Neurotoxin+ | K25-N27-K34 | 9.2Å - 6.6Å - 6.0Å [10.5Å – 7.5Å - 8.3Å] | [6] |
| *Leiurus quinquestriatus* | Chlorotoxin (PDB ID:1CHL) | Neurotoxin+ | K27-Y29-R36 | 7.0Å – 6.1Å - 9.7Å [8.4Å – 5.3Å – 9.7Å] | [7] |
| *Grammostola rosea* | Hanatoxin type 1 (PDB ID:1D1H) | Toxin ($K^+$ channel) + | K10-T12-K26 | 7.0Å – 7.2Å – 9.6Å [7.2Å – 8.6Å – 10.8Å] | [8] |
| *Drosophila melanogaster* | Drosomycin (PDB ID:1MYN)* | Antifungal Peptide | R6-S4-K38 | 12.2Å - 5.5Å - 8.8Å [12.8Å - 7.3Å - 7.2Å] | [9] |
| *Conus geographus* | Conotoxin GS (PDB ID:1AG7) | Neurotoxin+ | R21-Q25-R18 | 9.3Å - 5.0Å - 11.1Å [10.1Å - 8.1Å - 12.3Å] | [10] |
| *Conus magus* | Omega-Conotoxin MVIIA (PDB ID:1MVI) | Toxin (N-type, $Ca^{2+}$ channel) + | R10-S22-K7 | 10.4Å-4.1Å-9.1 Å [11.5 Å-5.7 Å-9.4 Å] | [11] |
| *Conus purpurascens* | Kappa Conotoxin PVIIA (PDB ID:1AV3) | Toxin ($K^+$ channel) + | R22-N24-K19 | 9.9 Å-5.4 Å-9.6 Å [11.0 Å-7.4 Å-10.6 Å] | [12] |
| *Tachypleus tridentatus* | Tachystatin A ( PDB ID:1CIX) | Antimicrobial Peptide | R43-Q7-R30 | 11.5 Å-6.2 Å-12.9 Å [11.5 Å-5.3 Å-11.0 Å] | [13] |
| *Tachypleus tridentatus* | Tachystatin B (PDB ID:2DCV) | Antimicrobial Peptide | R26-N41-R39 | 9.9 Å-5.5 Å-12.9 Å [11.1 Å-4.6 Å-11.4 Å] | [14] |
| *Peirates turpis* | PTU-1 (PDB ID:1I26) | Toxin ($Ca^{2+}$ channel) + | K32-S27-R23 | 14.2 Å-4.9 Å-12.5 Å | [15] |

| | | | | | |
|---|---|---|---|---|---|
| | | | | [15.1 Å-4.5 Å-13.7 Å] | |
| *Agriosphodrus dohrni* | ADO1 (PDB ID:1LMR) | Toxin ($Ca^{2+}$ channel) + | R31-T24-R8 | 7.0 Å-8.8 Å-10.0 Å [8.4 Å-10.5 Å-11.7 Å] | [16] |
| *Amaranthus hypochondriacus* | Plant Protein (PDB ID:1HTX) | Alpha-Amylase Inhibitor | K4-S32-R7 | 9.0 Å-6.0 Å-9.7 Å [11.4 Å-5.0 Å-11.7 Å] | [17] |
| *Petunia x hybrida* | PhD1 (PDB ID:1N4N) | Plant Defensin | K18-N16-R39 | 13.4 Å-5.5 Å-10.0 Å [15.5 Å-4.8 Å-12.3 Å] | [18] |
| *Centruroides noxius* | CnErg1 Ergotoxin (PDB ID:1PX9) | HERG Channel Inhibitor+ | R10-Y17-K13 | 10.6 Å-8.3 Å-11.7 Å [13.6 Å-7.1 Å-11.2 Å] | [19] |
| *Mesobuthus eupeus* | I5A *Buthus Eupeus* (PDB ID:1SIS) | Insectotoxin+ | K25-N23-K14 | 9.8 Å-7.1 Å-9.3 Å [14.3 Å-7.3 Å-12.8 Å] | [20] |
| *Hadronyche infensa* | ACTX-Hi:OB4219 (PDB ID:1KQH) | Venom Toxin+ | R34-S24-K19 | 9.9 Å-6.5 Å-13.3 Å [12.4 Å-4.5 Å-12.0 Å] | [20] |
| *Psalmopoeus cambridgei* | PcFK1 (PDB ID:1X5V) | Venom Toxin+ | R25-Y11-R20 | 14.1 Å-4.8 Å-14.3 Å [16.3 Å-6.7 Å-13.3 Å] | [21] |
| *Chassalia parviflora* | | Antimicrobial Peptide | CPC clip motif not identified | | [22] |
| *Viola odorata* | Cycloviolacin O1 (PDB ID: 1DF6) | Defensive Peptide | CPC clip motif not identified | | [23] |
| *Asteropus sp.* | Asteropsin A (PDB ID: 2LQA) | Neurotoxin+ | CPC clip motif not identified | | [24] |
| *Mesembryanthemum crystallinum* | Antimicrobial peptide 1 (precursor) (NCBI ID: AAC19399) Φ | Antimicrobial peptide | K34-Y60-R58 | 10.8 Å-5.4 Å-7.0 Å [9.5 Å-8.2 Å-6.4 Å] | Unpublished |

| | | | | | |
|---|---|---|---|---|---|
| *Aceria tosichella* | Antimicrobial peptide 1, partial (NCBI ID:MDE48292.1) Φ | Antimicrobial peptide | K145-N146-R178 | 9.4 Å-3.7 Å-10.6 Å [10.3 Å-5.7 Å-11.6 Å] | Unpublished |
| *Callosobruchus maculatus* | Unnamed protein product (NCBI ID:VEN35376.1) Φ | Unknown | R64-Y53-K59 | 16.5 Å-7.8 Å-10.3 Å [15.6 Å-6.8 Å-9.9 Å] | N/A |
| *Dinothrombium tinctorium* | Hypothetical protein B4U79_17867 (NCBI ID:RWS16713.1) Φ | Unknown | R59-N30-R61 | 6.6 Å-5.8 Å-11.0 Å [7.1 Å-7.7 Å-11.1 Å] | N/A |
| *Trichogramma pretiosum* | Antimicrobial peptide Alo-2-like (NCBI ID: XP_014233229.1) Φ | Putative Antimicrobial peptide | R62-N49-K52 | 11.1 Å-5.0 Å-9.9 Å [9.9 Å-4.7 Å-9.9 Å] | N/A |
| *Ignelater luminosus* | Hypothetical protein ILUMI_21843 (NCBI ID: KAF2884324.1) Φ | Unknown | R57-Y44-K47 | 11.1 Å-5.3 Å-9.9 Å [11.2 Å-4.1 Å-9.8 Å] | N/A |
| *Trichogramma brassicae* | Unnamed protein product (NCBI ID: CAB0031014.1) Φ | Unknown | R52-Y41-R54 | 6.5 Å-7.7 Å-10.9 Å [9.3 Å-7.1 Å-11.1 Å] | N/A |
| *Agrilus planipennis* | Antimicrobial peptide 1-like isoform X2 (NCBI ID: XP_018321119.1) Φ | Putative Antimicrobial peptide | R102-Y88-R72 | 9.1 Å-5.8 Å-10.2 Å [9.3 Å-8.5 Å-11.5 Å] | N/A |
| *Neodiprion lecontei* | Uncharacterized protein LOC107222248 (NCBI ID: XP_015517007.2) Φ | Unknown | R128-Y115-K118 | 10.9 Å-4.9 Å-10.0 Å [9.9 Å-4.4 Å-8.8 Å] | N/A |
| *Brassicogethes aeneus* | Unnamed protein product (NCBI ID: CAH0563829.1) Φ | Unknown | CPC clip motif not identified | | N/A |

| | | | | | |
|---|---|---|---|---|---|
| *Akanthomyces lecanii* | Gurmarin-like inhibitors/Antifungal toxin (Uniprot ID: A0A168C5L6) Φ | Putative Antifungal toxin | K60-N43-K39 | 11.0 Å-6.3 Å-12.4 Å [11.3 Å-5.5 Å-12.0 Å] | N/A |
| *Cordyceps Javanica* | Antifungal peptide domain-containing protein (Uniprot ID: A0A545VVU1) Φ | Putative Antifungal peptide | K17-T24-K14 | 10.3 Å-9.4 Å-9.8 Å [8.9 Å-9.6 Å-8.5 Å] | N/A |
| *Cordyceps confragosa* | Uncharacterized protein (Uniprot ID: A0A179IJT7) Φ | Unknown | K117-N116-K98 | 6.8 Å-3.8 Å-9.9 Å [7.4 Å-4.5 Å-10.9 Å] | N/A |
| *Beauveria bassiana* | Uncharacterized protein (Uniprot ID: A0A0A2VUF6) Φ | Unknown | K37-T47-R31 | 9.6 Å-9.8 Å-10.3 Å [8.6 Å-7.5 Å-12.0 Å] | N/A |
| *Rosellinia necatrix* | Uncharacterized protein (Uniprot ID: A0A1S8A723) Φ | Unknown | CPC clip motif not identified | | N/A |
| ***Leptopilina heterotoma* knottins** | | | | | |
| *Leptopilina heterotoma* | *L. heterotoma* knottin 2 (NCBI ID: GHUQ01074597.1) Φ | Unknown | K61-Y59-R57 | 13.5 Å-6.6 Å-7.0 Å [13.4 Å-5.8 Å-7.7 Å] | N/A |
| *Leptopilina heterotoma* | *L. heterotoma* knottin 3 (NCBI ID: GHUQ01047008.1) Φ | Unknown | R50-Q49-K37 | 7.8 Å-3.8 Å-8.8 Å [8.1 Å-5.0 Å-7.8 Å] | N/A |
| *Leptopilina heterotoma* | *L. heterotoma* knottin 4 (NCBI ID: GHUQ01062822.1; GHUQ01062810.1) Φ | Unknown | CPC clip motif not identified | | N/A |
| *Leptopilina heterotoma* | *L. heterotoma* knottin 5 | Unknown | CPC clip motif not identified | | N/A |

| | | | | | |
|---|---|---|---|---|---|
| | (NCBI ID: GHUP01112277.1±; GHUP01112278.1) Φ | | | | |
| *Leptopilina heterotoma* | *L. heterotoma* knottin 6 (NCBI ID: GHUP01112276.1±; GHUP01112279.1) Φ | Unknown | CPC clip motif not identified | | N/A |
| *Leptopilina heterotoma* | *L. heterotoma* knottin 7 (NCBI ID: GHUP01112276.1±; GHUP01112277.1±) Φ | Unknown | K38-Q34-K27 | 16.7 Å-6.7 Å-14.3 Å [15.4 Å-7.7 Å-13.5 Å] | N/A |
| *Leptopilina heterotoma* | *L. heterotoma* knottin 8 (NCBI ID: GAJC01013378.1; GHUQ01006582.1) Φ | Unknown | K60-N58-K46 | 7.6 Å-6.6 Å-10.7 Å [9.1 Å-7.5 Å-12.7 Å] | N/A |
| *Leptopilina heterotoma* | *L. heterotoma* knottin 9 (NCBI ID: GHUQ01006580.1; GHUQ01006581.1; GHUP01140860.1; GHUP01140857.1) Φ | Unknown | K50-Y49-K60 | 10.6 Å-3.8 Å-7.2 Å [9.9 Å-4.6 Å-6.7 Å] | N/A |
| *Leptopilina heterotoma* | *L. heterotoma* knottin 10 (NCBI ID: GAJC01011443.1; GHUQ01051051.1) Φ | Unknown | R60-Y49-R62 | 6.3 Å-7.5 Å-10.7 Å [10.5 Å-6.5 Å-12.4 Å] | N/A |
| *Leptopilina heterotoma* | *L. heterotoma* knottin 11 (NCBI ID: GHUP01089975.1) Φ | Unknown | R58-Y47-R60 | 6.4 Å-7.4 Å-10.9 Å [8.7 Å-8.2 Å-11.3 Å] | N/A |
| *Leptopilina heterotoma* | *L. heterotoma* knottin 12 | Unknown | R57-Y46-R59 | 6.4 Å-7.4 Å-11.2 Å [7.9 Å-6.6 Å-13.0 Å] | N/A |

| | | | | | |
|---|---|---|---|---|---|
| | (NCBI ID: GHUQ01026197.1; GHUP01112307.1) Φ | | | | |
| *Leptopilina heterotoma* | *L. heterotoma* knottin 13 (NCBI ID: GHUQ01062808.1) Φ | Unknown | R37-T38-K47 | 12.6 Å-3.8 Å-10.8 Å [14.1 Å-5.8 Å-9.7 Å] | N/A |
| *Leptopilina heterotoma* | *L. heterotoma* knottin 14 (NCBI ID: GHUP01093382.1; GHUP01093384.1) Φ | Unknown | R37-T38-K47 | 12.1 Å-3.8 Å-10.9 Å [14.0 Å-6.3 Å-9.7 Å] | N/A |
| *Leptopilina heterotoma* | *L. heterotoma* knottin 15 (NCBI ID: GHUP01011997.1) Φ | Unknown | R31-N32-R28 | 9.8 Å-3.7 Å-12.3 Å [11.1 Å-3.7 Å-10.8 Å] | N/A |
| *Leptopilina heterotoma* | *L. heterotoma* knottin 16 (NCBI ID: GAJC01018499.1; GHUQ01075921.1; GHUP01000498.1; GHUP01000497.1) Φ | Unknown | K36-T57-H33 | 10.3 Å-5.5 Å-10.8 Å [9.1 Å-7.9 Å-10.9 Å] | N/A |
| *Leptopilina heterotoma* | *L. heterotoma* knottin 17 (NCBI ID: GAJC01009090.1; GHUQ01090262.1; GHUP01053005.1) Φ | Unknown | R61-N31-R34 | 9.2 Å-5.9 Å-9.7 Å [12.4 Å-7.5 Å-13.3 Å] | N/A |
| ***Leptopilina boulardi* knottins** | | | | | |
| *Leptopilina boulardi* | *L. boulardi* knottin 1 (NCBI ID: GAJA01018727.1; GISX01146955.1; GITC01024163.1) Φ | Unknown | K27-N29-K25 | 6.6 Å-5.6 Å-12.1 Å [6.6Å-6.5 Å-13.0 Å] | N/A |

| | | | | | |
|---|---|---|---|---|---|
| *Leptopilina boulardi* | *L. boulardi* knottin 2 (NCBI ID: GAJA01018424.1; GISX01025604.1; GISX01025592.1; GITC01178462.1; GITC01178474.1; GITC01178472.1; GITC01178467.1; GITC01178471.1; GITC01178466.1; GITC01178468.1) Φ | Unknown | R59-Y44-H46 | 10.7 Å-5.8 Å-6.8 Å [11.3 Å-5.3 Å-6.5 Å] | N/A |
| *Leptopilina boulardi* | *L. boulardi* knottin 3 (NCBI ID: GAJA01013374.1) | Unknown | CPC clip motif not identified | | N/A |
| *Leptopilina boulardi* | *L. boulardi* knottin 4 (NCBI ID: GAJA01001863.1; GGGI01004962.1) Φ | Unknown | K50-Q35-R32 | 11.6 Å-7.1 Å-10.4 Å [12.6 Å-9.3 Å-8.9 Å] | N/A |
| *Leptopilina boulardi* | *L. boulardi* knottin 5 (NCBI ID: GISX01146961.1) Φ | Unknown | K57-Y46-R59 | 6.5 Å-7.6 Å-10.9 Å [8.8 Å-7.4 Å-11.1 Å] | N/A |
| *Leptopilina boulardi* | *L. boulardi* knottin 6 (NCBI ID: GISX01025601.1) Φ | Unknown | K28-N24-R44 | 7.2 Å-6.1 Å-13.0 Å [8.0 Å-5.9 Å-13.7 Å] | N/A |
| *Leptopilina boulardi* | *L. boulardi* knottin 7 (NCBI ID: GISX01146968.1) Φ | Unknown | R63-Y52-R65 | 6.5 Å-7.5 Å-11.4 Å [9.8 Å-8.0 Å-13.5 Å] | N/A |
| *Leptopilina boulardi* | *L. boulardi* knottin 8 (NCBI ID: GISX01046436.1) Φ | Unknown | R29-S35-K38 | 15.4 Å-9.0 Å-10.1 Å [13.7 Å-9.4 Å-10.0 Å] | N/A |

| | | | | | |
|---|---|---|---|---|---|
| *Leptopilina boulardi* | *L. boulardi* knottin 9 (NCBI ID: GISX01016500.1; GITC01073258.1) Φ | Unknown | K47-N48-K51 | 12.8 Å-3.7 Å-9.7 Å [11.5 Å-5.2 Å-10.1 Å] | N/A |
| *Leptopilina boulardi* | *L. boulardi* knottin 10 (NCBI ID: GISX01112026.1; GITC01104371.1; GGGI01012121.1) Φ | Unknown | R60-Y49-R62 | 6.7 Å-7.6 Å-12.1 Å [8.3 Å-6.8 Å-10.8 Å] | N/A |
| *Leptopilina boulardi* | *L. boulardi* knottin 11 (NCBI ID: GISX01151661.1; GISX01151658.1; GITC01143021.1) Φ | Unknown | R83-N52-R68 | 7.0 Å- 7.6 Å-11.6 Å [10.7 Å-10.2 Å-14.6 Å] | N/A |
| *Leptopilina boulardi* | *L. boulardi* knottin 12 (NCBI ID: GISX01151653.1; GITC01143015.1) Φ | Unknown | R60-Q74-R70 | 11.8 Å-6.8 Å-12.9 Å [12.9 Å-4.4 Å-14.6 Å] | N/A |
| *Leptopilina boulardi* | *L. boulardi* knottin 13 (NCBI ID: GISX01112051.1; GITC01104365.1; GGGI01006767.1) Φ | Unknown | R51-Y53-R45 | 11.4 Å-5.4 Å-10.6 Å [12.3 Å-7.1 Å-10.3 Å] | N/A |
| *Leptopilina boulardi* | *L. boulardi* knottin 14 (NCBI ID: GISX01121040.1; GITC01206261.1) Φ | Unknown | H42-N38-K71 | 6.6 Å-4.2Å-7.8 Å [7.8 Å-3.3 Å-8.7 Å] | N/A |
| *Leptopilina boulardi* | *L. boulardi* knottin 15 (NCBI ID: GISX01121036.1; GISX01121038.1; | Unknown | H31-N27-K60 | 6.6 Å-4.6 Å-8.2 Å [7.6 Å-4.4 Å-8.7 Å] | N/A |

| | | | | | |
|---|---|---|---|---|---|
| | GITC01206267.1) Φ | | | | |
| *Leptopilina boulardi* | *L. boulardi* knottin 16 (NCBI ID: GISX01056433.1; GITC01031425.1) Φ | Unknown | R32-N33-R60 | 6.3 Å-3.8 Å-8.8 Å [8.5 Å-3.6 Å-8.8 Å] | N/A |
| *Leptopilina boulardi* | *L. boulardi* knottin 17 (NCBI ID: GISX01112052.1) Φ | Unknown | R51-Y52-R68 | 7.9 Å-3.7 Å-8.3 Å [6.4 Å-5.6 Å-8.3 Å] | N/A |
| *Leptopilina boulardi* | *L. boulardi* knottin 18 (NCBI ID: GITC01044290.1) Φ | Unknown | K48-Q49-K36 | 8.6 Å-3.8 Å-9.9 Å [8.5 Å-4.8 Å-11.0 Å] | N/A |
| *Leptopilina boulardi* | *L. boulardi* knottin 19 (NCBI ID: GITC01089342.1) Φ | Unknown | R63-Y52-R65 | 6.3 Å-7.6 Å-10.0 Å [9.9 Å-7.7 Å-12.3 Å] | N/A |
| *Leptopilina boulardi* | *L. boulardi* knottin 20 (NCBI ID: GITC01142054.1) | Unknown | CPC clip motif not identified | | N/A |
| *Leptopilina boulardi* | *L. boulardi* knottin 21 (NCBI ID: GITC01158992.1) Φ | Unknown | R57-T68-R59 | 6.8 Å-7.5 Å-14.0 Å [8.9 Å-5.7 Å-14.1 Å] | N/A |
| *Leptopilina boulardi* | *L. boulardi* knottin 22 (NCBI ID: GGGI01003630.1) Φ | Unknown | K59-T45-K49 | 11.0 Å-7.6 Å-12.9 Å [13.9 Å-9.0 Å-13.9 Å] | N/A |
| ***Ganaspis spp.*** **knottins** | | | | | |
| *Ganaspis hookeri* | *G. hookeri* knottin 1 (NCBI ID: GAIW01014505.1) Φ | Unknown | R60-Y49-R62 | 6.3 Å-7.5 Å-10.7 Å [10.5 Å-6.5 Å-12.4 Å] | N/A |
| *Ganaspis hookeri* | *G. hookeri* knottin 2 (NCBI ID: GAIW01021714.1) Φ | Unknown | K34-N44-K28 | 9.5 Å-9.9 Å-10.5 Å [9.0 Å-7.7 Å-10.9 Å] | N/A |

| | | | | | |
|---|---|---|---|---|---|
| *Ganaspis hookeri* | *G. hookeri* knottin 3 (NCBI ID: GAIW01010507.1) Φ | Unknown | K38-S60-K33 | 15.7 Å-7.9 Å-8.7 Å [15.1 Å-7.4 Å-8.5 Å] | N/A |
| *Ganaspis hookeri* | *G. hookeri* knottin 4 (NCBI ID: GAIW01013268.1) Φ | Unknown | R65-T49-K63 | 6.6 Å-7.3 Å-7.3 Å [6.8 Å-5.2 Å-8.3 Å] | N/A |
| *Ganaspis hookeri* | *G. hookeri* knottin 5 (NCBI ID: GAIW01013559.1) Φ | Unknown | K31-S30-K15 | 11.7 Å-3.8 Å-8.7 Å [12.8 Å-4.2 Å-8.8 Å] | N/A |
| *Ganaspis hookeri* | *G. hookeri* knottin 6 (NCBI ID: GAIW01004791.1) Φ | Unknown | R57-Q58-K53 | 11.2 Å-3.8 Å-14.3 Å [10.5 Å-7.9 Å-15.3 Å] | N/A |
| *Ganaspis brasiliensis* | *G. brasiliensis* knottin 1 (In house ID: g19460.t1) Φ | Unknown | H62-T64-K54 | 6.2 Å-6.4 Å-10.8 Å [9.8 Å-6.3 Å-12.6 Å] | N/A |
| *Ganaspis brasiliensis* | *G. brasiliensis* knottin 2 ( In house ID: g14381.t1) Φ | Unknown | K62-Q54-K39 | 7.9 Å-6.1 Å-7.1 Å [8.7 Å-9.4 Å-7.4 Å] | N/A |

A

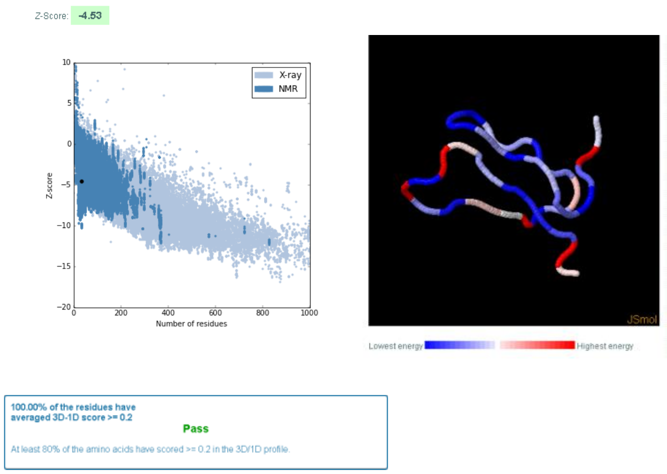
Z-Score: -4.53
X-ray
NMR
Z-score
Number of residues
JSmol
Lowest energy
Highest energy
100.00% of the residues have averaged 3D-1D score >= 0.2
Pass
At least 80% of the amino acids have scored >= 0.2 in the 3D/1D profile.

B

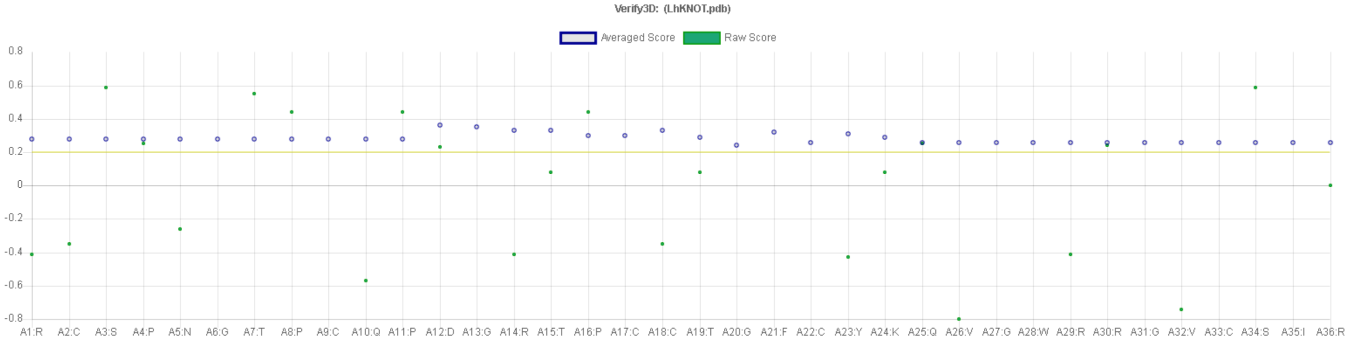
Verify3D: (LhKNOT.pdb)
Averaged Score
Raw Score
A1:R A2:C A3:S A4:P A5:N A6:G A7:T A8:P A9:C A10:Q A11:P A12:D A13:G A14:R A15:T A16:P A17:C A18:C A19:T A20:G A21:F A22:C A23:Y A24:K A25:Q A26:V A27:G A28:W A29:R A30:R A31:G A32:V A33:C A34:S A35:I A36:R

**C**

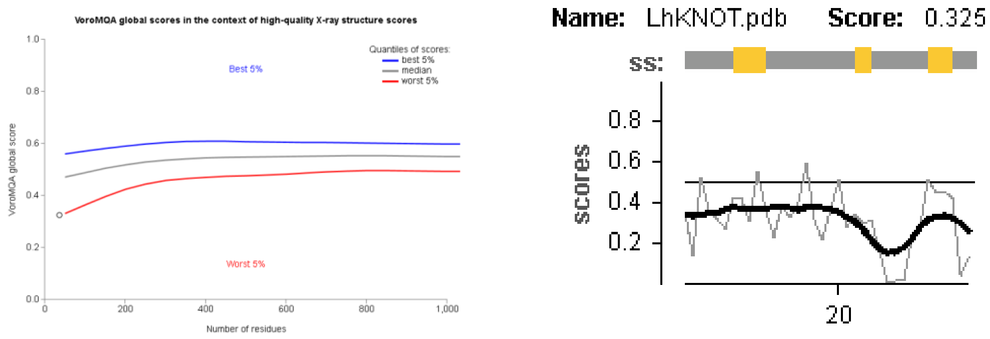


**Figure S1. (A)** Prosa-web evaluation profile. Z-score of the model is represented as a black dot on a backdrop of Z-scores of known structures from the pdb (left) and visualization of the three-dimensional model LhKNOT with residues colored from blue to red in the order of increasing residue energy (right). **(B)** Verify3d evaluation profile. The plot shows the averaged and raw 1D-3D scores for LhKNOT and panel on top lists the percentage of residues that scored an average of 0.2 or above. A passing model has at least 80% of its residues scoring above 0.2. **(C)** VoroMQA evaluation profile. VoroMQA global scores in the context of high-quality X-ray structure scores (left) and plot of local score and overall score for LhKNOT (right).

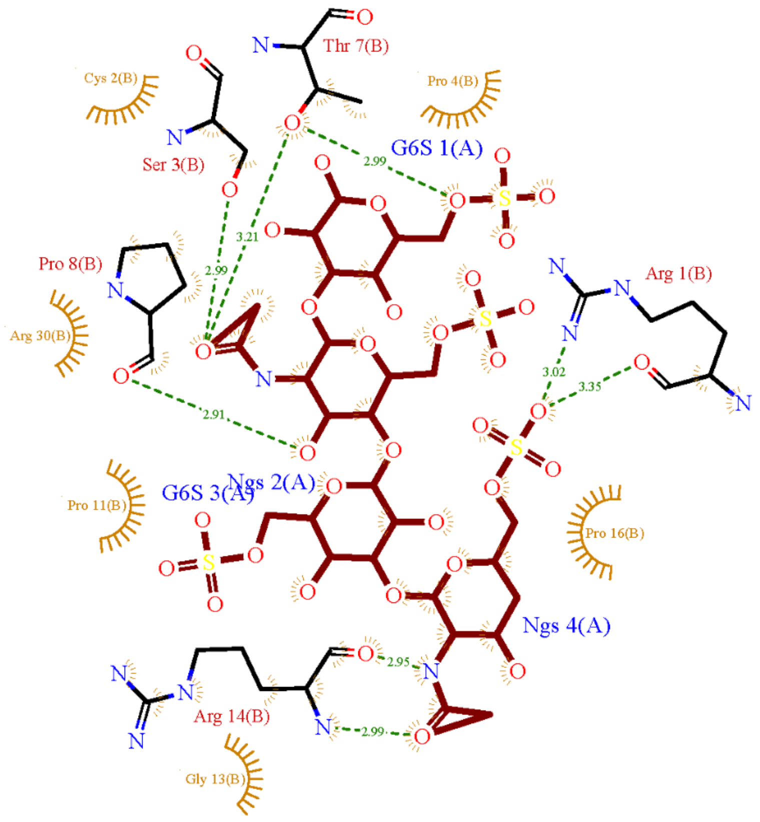


**Figure S2**. Ligplot showing interactions of LhKNOT with keratan sulfate (PDB: 1KES) include the CPC Clip residues (R1-S3-R14). Green dashed lines depict hydrogen bond interactions.

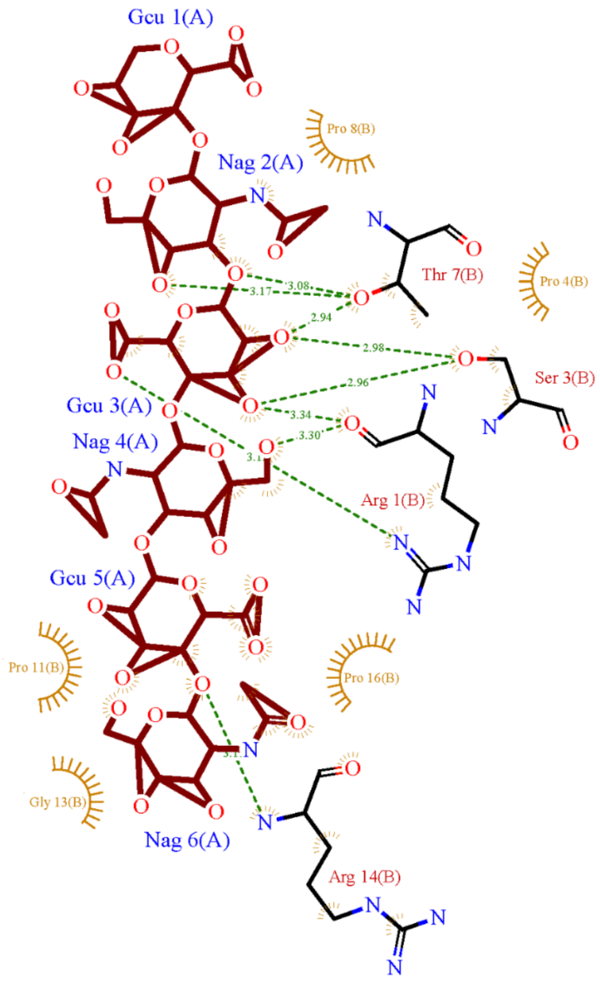


**Figure S3**. Ligplot showing interactions of LhKNOT with hyaluronic acid (PDB: 1HYA) include the CPC Clip residues (R1-S3-R14). Green dashed lines depict hydrogen bond interactions.

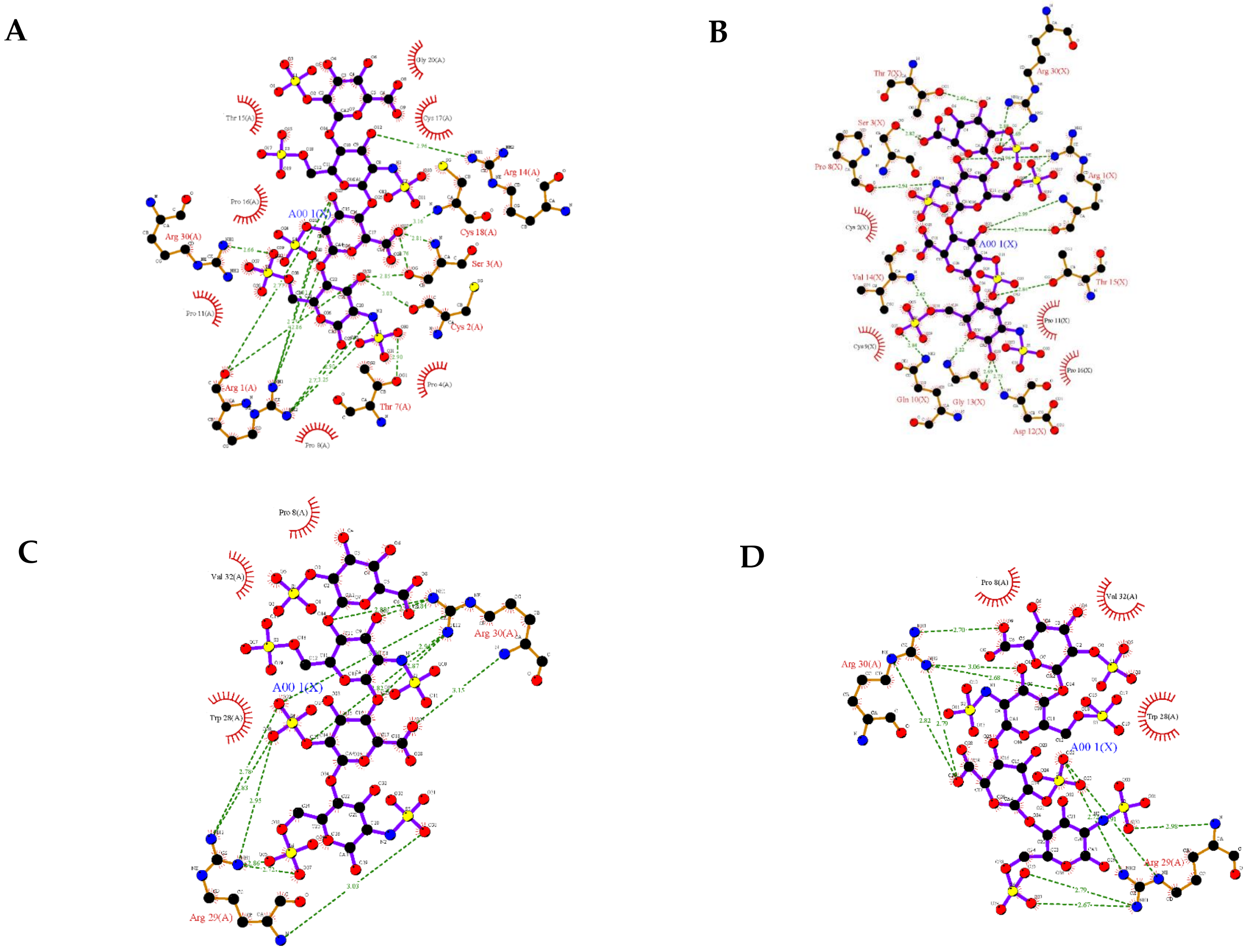


**Figure S4**.**(A)** Ligplot showing interactions of wild type LhKNOT interacting with heparin via with the primary CPC Clip (R1-S3-R14). (**B)** Ligplot showing interactions of mutant R14A LhKNOT interacting with heparin via a secondary CPC Clip (R1-S3-R30). (**C)** Ligplot

showing interactions of the double mutant (R14A-R1A) LhKNOT interacting with heparin via non-specific basic residues, no CPC clip identified. (**D)** Ligplot showing interactions of the triple mutant (R14A-S3A-R1A) LhKNOT interacting with heparin via non-specific basic residues, , no CPC clip identified. Green dashed lines depict hydrogen bond interactions.

## References


1. Heavner ME, Ramroop J, Gueguen G, Ramrattan G, Dolios G, Scarpati M, et al. Novel Organelles with Elements of Bacterial and Eukaryotic Secretion Systems Weaponize Parasites of *Drosophila*. Curr Biol. 2017;27: 2869-2877.e6. doi: S0960-9822(17)31028-X [pii].

2. Barbault F, Landon C, Guenneugues M, Meyer JP, Schott V, Dimarcq JL, et al. Solution structure of Alo-3: a new knottin-type antifungal peptide from the insect *Acrocinus longimanus*. Biochemistry. 2003;42: 14434-14442. doi: 10.1021/bi035400o [doi].

3. Gao GH, Liu W, Dai JX, Wang JF, Hu Z, Zhang Y, et al. Solution structure of PAFP-S: a new knottin-type antifungal peptide from the seeds of *Phytolacca americana*. Biochemistry. 2001;40: 10973-10978. doi: bi010167k [pii].

4. Loo S, Kam A, Xiao T, Tam JP. Bleogens: Cactus-Derived Anti-*Candida* Cysteine-Rich Peptides with Three Different Precursor Arrangements. Front Plant Sci. 2017;8: 2162. doi: 10.3389/fpls.2017.02162 [doi].

5. Reily MD, Holub KE, Gray WR, Norris TM, Adams ME. Structure-activity relationships for P-type calcium channel-selective omega-agatoxins. Nat Struct Biol. 1994;1: 853-856.

6. Fletcher JI, Smith R, O'Donoghue SI, Nilges M, Connor M, Howden ME, et al. The structure of a novel insecticidal neurotoxin, omega-atracotoxin-HV1, from the venom of an Australian funnel web spider. Nat Struct Biol. 1997;4: 559-566.

7. Lippens G, Najib J, Wodak SJ, Tartar A. NMR sequential assignments and solution structure of chlorotoxin, a small scorpion toxin that blocks chloride channels. Biochemistry. 1995;34: 13-21. doi: 10.1021/bi00001a003 [doi].

8. Takahashi H, Kim JI, Min HJ, Sato K, Swartz KJ, Shimada I. Solution structure of hanatoxin1, a gating modifier of voltage-dependent K(+) channels: common surface features of gating modifier toxins. J Mol Biol. 2000;297: 771-780. doi: S0022-2836(00)93609-X [pii].

9. Landon C, Pajon A, Vovelle F, Sodano P. The active site of drosomycin, a small insect antifungal protein, delineated by comparison with the modeled structure of Rs-AFP2, a plant antifungal protein. J Pept Res. 2000;56: 231-238.

10. Hill JM, Alewood PF, Craik DJ. Solution structure of the sodium channel antagonist conotoxin GS: a new molecular caliper for probing sodium channel geometry. Structure. 1997;5: 571-583. doi: S0969-2126(97)00212-8 [pii].

11. Nielsen KJ, Thomas L, Lewis RJ, Alewood PF, Craik DJ. A consensus structure for omega-conotoxins with different selectivities for voltage-sensitive calcium channel subtypes: comparison of MVIIA, SVIB and SNX-202. J Mol Biol. 1996;263: 297-310. doi: S0022-2836(96)90576-8 [pii].

12. Scanlon MJ, Naranjo D, Thomas L, Alewood PF, Lewis RJ, Craik DJ. Solution structure and proposed binding mechanism of a novel potassium channel toxin kappa-conotoxin PVIIA. Structure. 1997;5: 1585-1597. doi: S0969-2126(97)00307-9 [pii].

13. Fujitani N, Kawabata S, Osaki T, Kumaki Y, Demura M, Nitta K, et al. Structure of the antimicrobial peptide tachystatin A. J Biol Chem. 2002;277: 23651-23657. doi: 10.1074/jbc.M111120200 [doi].

14. Fujitani N, Kouno T, Nakahara T, Takaya K, Osaki T, Kawabata S, et al. The solution structure of horseshoe crab antimicrobial peptide tachystatin B with an inhibitory cystine-knot motif. J Pept Sci. 2007;13: 269-279. doi: 10.1002/psc.846 [doi].

15. Bernard C, Corzo G, Mosbah A, Nakajima T, Darbon H. Solution structure of Ptu1, a toxin from the assassin bug *Peirates turpis* that blocks the voltage-sensitive calcium channel N-type. Biochemistry. 2001;40: 12795-12800. doi: bi015537j [pii].

16. Bernard C, Corzo G, Adachi-Akahane S, Foures G, Kanemaru K, Furukawa Y, et al. Solution structure of ADO1, a toxin extracted from the saliva of the assassin bug, *Agriosphodrus dohrni*. Proteins. 2004;54: 195-205. doi: 10.1002/prot.10513 [doi].

17. Martins JC, Enassar M, Willem R, Wieruzeski JM, Lippens G, Wodak SJ. Solution structure of the main alpha-amylase inhibitor from amaranth seeds. Eur J Biochem. 2001;268: 2379-2389. doi: ejb2118 [pii].

18. Janssen BJC, Schirra HJ, Lay FT, Anderson MA, Craik DJ. Structure of *Petunia hybrida* defensin 1, a novel plant defensin with five disulfide bonds. Biochemistry. 2003;42: 8214-8222. doi: 10.1021/bi034379o [doi].

19. Frenal K, Xu C, Wolff N, Wecker K, Gurrola GB, Zhu S, et al. Exploring structural features of the interaction between the scorpion toxinCnErg1 and ERG K+ channels. Proteins. 2004;56: 367-375. doi: 10.1002/prot.20102 [doi].

20. Lomize AL, Maiorov VN, Arsen'ev AS. Determination of the spatial structure of insectotoxin 15A from *Buthus erpeus* by (1)H-NMR spectroscopy data. Bioorg Khim. 1991;17: 1613-1632.

21. Pimentel C, Choi S, Chagot B, Guette C, Camadro J, Darbon H. Solution structure of PcFK1, a spider peptide active against *Plasmodium falciparum*. Protein Sci. 2006;15: 628-634. doi: ps.051860606 [pii].

22. Koltay A, Daly NL, Gustafson KR, Craik DJ. Structure of Circulin B and Implications for Antimicrobial Activity of the Cyclotides. International Journal of Peptide Research and Therapeutics. 2005;11: 99-106. doi: 10.1007/s10989-004-1722-2.

23. Craik DJ, Daly NL, Bond T, Waine C. Plant cyclotides: A unique family of cyclic and knotted proteins that defines the cyclic cystine knot structural motif. J Mol Biol. 1999;294: 1327-1336. doi: 10.1006/jmbi.1999.3383.

24. Li H, Bowling JJ, Fronczek FR, Hong J, Jabba SV, Murray TF, et al. Asteropsin A: an unusual cystine-crosslinked peptide from porifera enhances neuronal Ca2+ influx. Biochim Biophys Acta. 2013;1830: 2591-2599. doi: 10.1016/j.bbagen.2012.11.015.
